# Rare renal cell carcinoma with haemangioblastoma-like features and leiomyomatous stroma: report of a unique case with *TSC2* and *SETD2* variations

**DOI:** 10.1186/s12957-022-02844-3

**Published:** 2022-12-13

**Authors:** Jixia Kong, Juan Tao, Qimin Wang, Qingfu Zhang, Liying Yin

**Affiliations:** 1 Department of Pathology, the Second Affiliated Hospital of Dalian Medical University, Dalian, 116027 Liaoning China; 2 Department of Pathology, the First Affiliated Hospital of China Medical University, Shenyang, 110001 Liaoning China

**Keywords:** Renal cell carcinoma, Haemangioblastoma, Leiomyomatous stroma, *TSC2*, *SETD2*

## Abstract

**Background:**

Renal cell carcinoma (RCC) with haemangioblastoma-like characteristics is a type of RCC reported in recent years. RCC with (angio) leiomyomatous stroma (RCCLMS) was included as a provisional entity of the 2016 World Health Organization (WHO) classification. RCC with haemangioblastoma-like characteristics and leiomyomatous stroma is extremely rare. This is the first report of a rare tumour harbouring *TSC2* and *SETD2* variations.

**Case presentation:**

The patient was a 38-year-old woman who presented with discomfort in the area of her right kidney. Ultrasound and enhanced CT showed a right renal mass, and clear cell renal cell carcinoma (CCRCC) was suspected; hence, robot-assisted laparoscopic nephron-sparing partial nephrectomy was performed. Gross examination revealed a well-circumscribed tumour measuring 2.0 cm × 1 cm × 0.7 cm under the renal capsule adjacent to the stripping edge that was greyish yellow and greyish red in colour. Histologic examination showed that the tumour consisted of three different structures: a CCRCC-like region, a haemangioblastoma-like region, and a focal leiomyomatous stroma component. Based on immunohistochemistry, the CCRCC-like region was diffusely strongly positive for AE1/AE3, vimentin, CAIX, PAX8, PAX2, CK7, and CAM5.2, partly positive for HNF1α, and negative for CD10, α-inhibin, NSE, S-100, CD34, and TFE3. The haemangioblastoma-like area was diffusely positive for vimentin, CAIX; partly positive for PAX8, PAX2, *α*-inhibin, and S-100; mostly positive for NSE; and slightly positive for HNF1α; the CD34 staining highlighted the complex capillary network. The Ki67 index was approximately 1–2% in the two above areas, and the leiomyomatous stroma was strongly positive for SMA. The whole-exon sequencing (WES) showed *TSC2* and *SETD2* variations. There was no progression after 18 months of follow-up.

**Conclusion:**

We report for the first time a unique case of RCC with haemangioblastoma-like features and leiomyomatous stroma accompanied by rare molecular abnormalities. Whether this is a new tumour entity or a variant of clear cell carcinoma remains to be determined. The biological behaviour and clinical characteristics need to be further examined.

## Background

Renal cell carcinoma (RCC) with haemangioblastoma-like characteristics is a type of RCC reported in recent years. It was first reported by Montironi R. et al. in 2014, regarding a tumour with special morphological characteristics, which included RCC and haemangioblastoma-like characteristics simultaneously [1]. It is extremely rare clinically. A thorough literature search revealed only 4 cases in the English literature [1–3] and 2 cases in the Chinese literature [4]. However, it was not introduced as a novel, morphologically defined RCC entity by the 2022 World Health Organization (WHO) classification of tumours of the urinary system and male genital organs [5]. In the 2016 WHO classification of renal epithelial neoplasia, RCC with (angio) leiomyomatous stroma (RCCLMS) was included as a provisional entity [6]. RCCLMS was once named adenomatoid tumour of renal vascular smooth muscle, RCC with significant angioleiomyomatoid, hyperplasia, and RCC with smooth muscle stroma, among others [7–9]. RCC with haemangioblastoma-like characteristics and leiomyomatous stroma is extremely rare. Due to the limited number of cases, its clinicopathological characteristics and significance, as well as molecular pathological changes, remain unclear. In this article, we first report a rare tumour that harbours *TSC2* and *SETD2* variations. Given the unique histomorphological characteristics of the tumour, it might be a new tumour entity or a variant of clear cell renal cell carcinoma (CCRCC).

## Case presentation

**Fig. 1:**
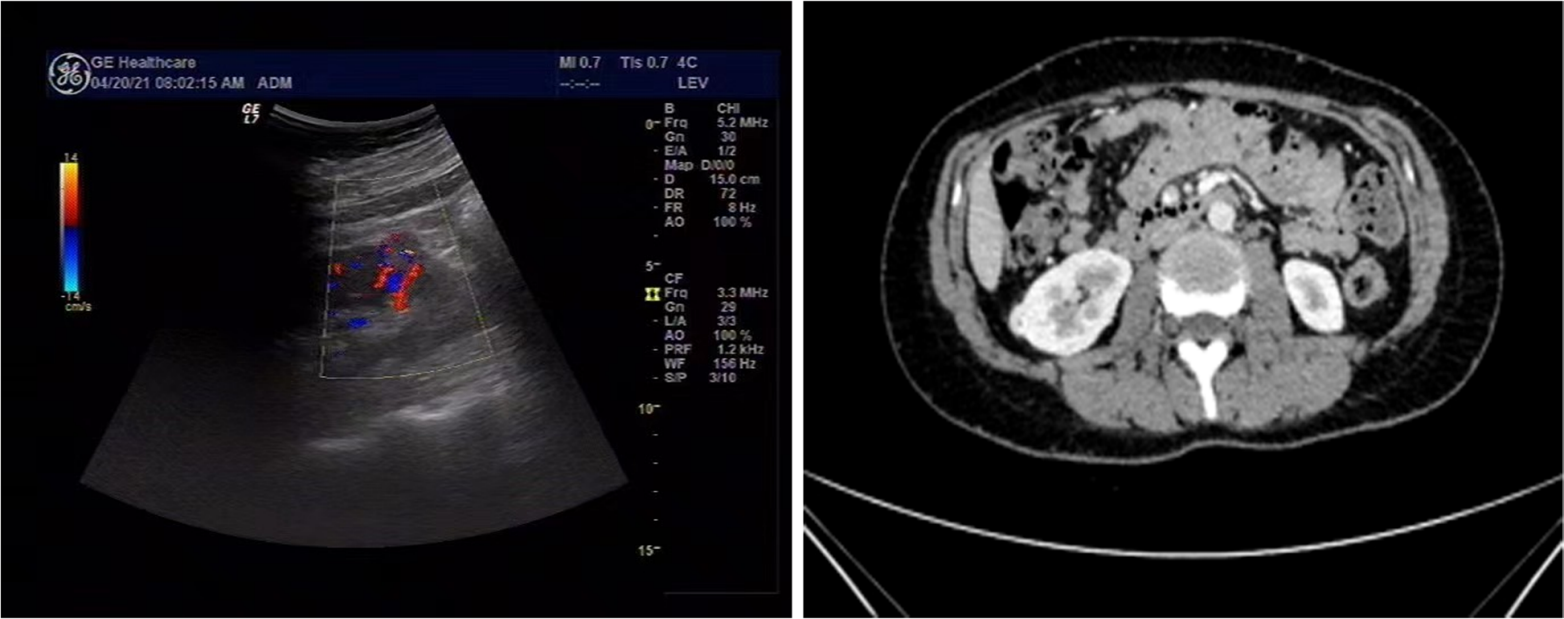
Imaging characteristics of the tumour. **A** Ultrasound image of this case. Ultrasound showed an isoechoic area at the lower pole of the right kidney, with a size of 1.6 × 1.3 cm, a clear boundary, and an uneven echo; it was slightly convex locally, and the blood flow signal could be seen. **B** Enhanced CT of the lower abdomen displayed a solid space occupying the right kidney

**Fig. 2:**
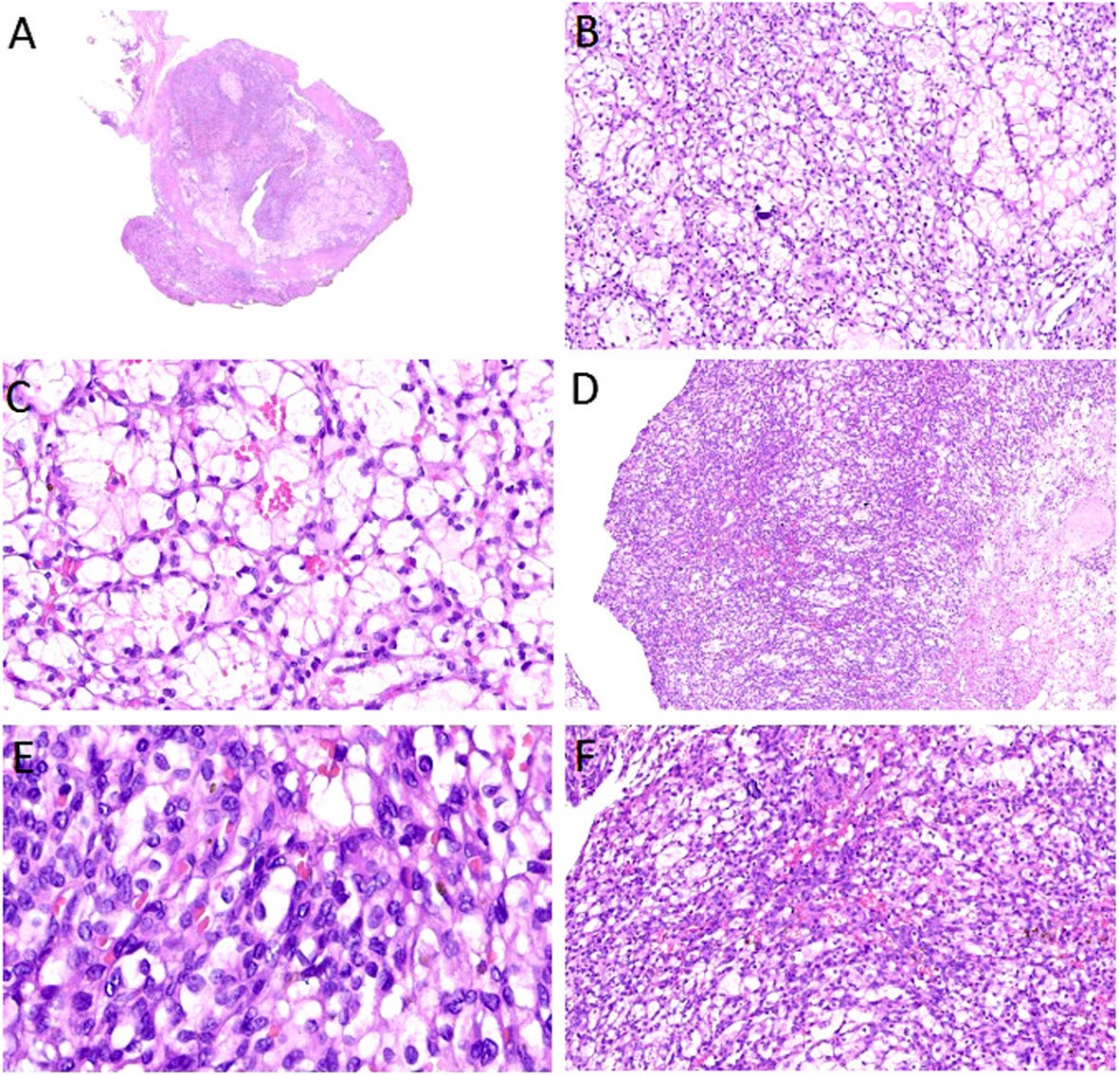
Morphological characteristics of the tumour. H&E staining. **A** Lower magnification showing a clear boundary, with thick fibromuscular tissue separated from normal renal tissue. (Original magnification ×6). **B** Tumour cells were arranged in acinar or glandular tubular shapes, with homogeneous pink staining in a few acinar cavities. The cell cytoplasm was transparent or slightly eosinophilic, and the boundary was vivid. (Original magnification ×100). **C** Most of the nuclei were located at the base, and some of them were irregular in shape; nucleoli could be seen in some cells. (Original magnification ×200). **D** Among the cancer cells, there were oval or polygonal cells with a flaky distribution and an obvious capillary network. Some areas are loose and edematous and devoid of cells, with a rich mesh of capillaries. (Original magnification ×40). **E** Tumour cells varied in size and possessed a pale or eosinophilic cytoplasm that sometimes contained sharply delineated fine vacuoles and hyaline globules. (Original magnification ×400). **F** Focal haemorrhage, haemosiderin deposition, and psammoma bodies were observed. Transition between haemangioblastoma-like area and CCRCC-like area could be seen. (Original magnification ×100)

A 38-year-old Chinese female who visited the hospital for physical examination due to discomfort in the area of her right kidney, and a solid mass in the right kidney was found for 4 months. The patient had microinvasive adenocarcinoma of the left lung 4 years prior, without obvious abnormalities in the right lung and other lobes of the left lung. The patient did not smoke and had no family history of major diseases. No obvious abnormality was found by laboratory examination. Ultrasound examination showed an isoechoic area at the lower pole of the right kidney, with a size of 1.6 × 1.3 cm, a clear boundary, and an uneven echo; it was slightly convex locally, and the blood flow signal could be seen (Fig. 1A). Enhanced CT of the lower abdomen revealed a solid space occupying the right kidney, and CCRCC was considered (Fig. 1B). Based on imaging findings, robot-assisted laparoscopic nephron-sparing partial nephrectomy was performed. Grossly, the tumour was a well-circumscribed lesion measuring 2.0 cm × 1.0 cm × 0.7 cm under the renal capsule, with a greyish yellow and greyish red colour. The tumour was submitted for histologic examination. Histologically, the tumour consisted of three different structures: a CCRCC-like region, a haemangioblastoma-like region, and a focal leiomyomatous stroma component (Fig. 2A). The CCRCC-like region accounted for approximately 60%, and the tumour cells were arranged in acinar or glandular tubular shapes, with homogeneous pink staining in a few acinar cavities but without papillary architecture. The cell cytoplasm was transparent or slightly eosinophilic, and the boundary was vivid (Fig. 2B). Most of the nuclei were located at the base, and some were irregular in shape. Nucleoli could be seen in some cells (WHO/ISUP grades 1 to 2, Fig. 2C). Among the cancer cells, we also observed oval or polygonal cells with a flaky distribution and obvious capillary network. The tumour cells varied in size and had a pale or eosinophilic cytoplasm that sometimes contained sharply delineated fine vacuoles and hyaline globules. The stroma was leiomyomatous, mainly located around the tumour; some areas were loose and edematous and devoid of cells, with a rich mesh of capillaries (Fig. 2D). The tumour cell nuclei with inconspicuous nucleoli showed light-moderate nuclear atypia and pleomorphism. Neither mitotic figures nor necrosis was present (Fig. 2E). Focal haemorrhage, haemosiderin deposition, and psammoma bodies were observed. Some areas showed prominent delicate vascularity, such as a haemangioblastoma, which transitioned with the CCRCC-like area (Fig. 2F).

**Fig. 3:**
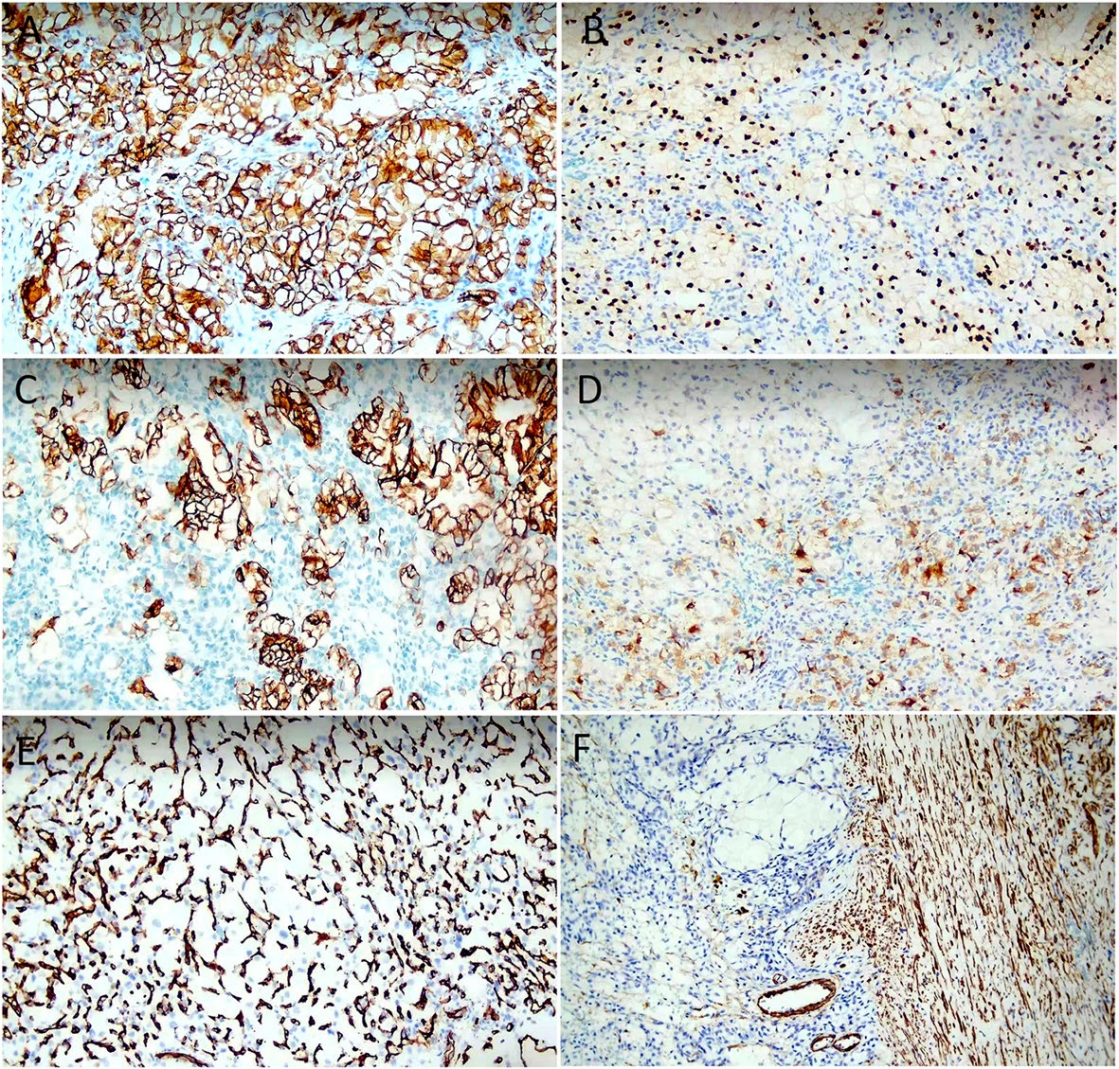
Immunohistochemical staining (original magnification ×200). **A** CAIX was diffusely strongly positive in the CCRCC-like region and haemangioblastoma-like region. **B** PAX8 was diffusely strongly positive in the CCRCC-like region and partly positive in the haemangioblastoma-like area. **C** CK7 was diffusely strongly in the CCRCC-like region. **D** *α*-inhibin was partly positive in the haemangioblastoma-like area. **E** CD34 staining in the complex capillary network. **F** SMA was diffusely positive in the leiomyomatous stroma

Immunohistochemical results showed that the CCRCC-like region was diffusely strongly positive for AE1/AE3, vimentin, CAIX (Fig. 3A), PAX8 (Fig. 3B), PAX2, CK7 (Fig. 3C), and CAM5.2, partly positive for HNF1α, and negative for CD10, α-inhibin, NSE, S-100, CD34, and TFE3. The haemangioblastoma-like region was diffusely positive for vimentin and CAIX; partly positive for PAX8, PAX2, α-inhibin (Fig. 3D), and S-100; mostly positive for NSE; and slightly positive for HNF1α. CD34 staining highlighted the complex capillary network (Fig. 3E). The Ki67 index was approximately 1–2% in these two areas. SMA was strongly positive in the leiomyomatous stroma (Fig. 3F). The whole-exon sequencing (WES) results of the tumour sample showed missense single-nucleotide variations (SNVs) in *TSC2* (NM_000548: exon 4:c.T311C:p. L104P) and *SETD2* (NM_014159:exon 3:c.C721G:p.P241A). The patient recovered well and was discharged after surgery. She has not received other treatments after the operation. She was in good condition and had no evidence of recurrence or metastasis at the 18-month follow-up after the operation.

## Discussion

Through a comprehensive literature review, a total of 7 cases of haemangioblastoma-like RCCs, including the case in our study, are summarized in Table 1. All cases involved adults aged 32–75 years, including 2 males and 5 females, affecting both the left and right kidneys. None of the patients had tuberous sclerosis syndrome or VHL syndrome. Among the cases reported in the literature, 6 patients had a good prognosis without follow-up treatment. Renal space occupation was mostly found during routine physical examination. Only 2 patients had clinical symptoms and a medical history (cases 3 and 7). The size of the tumour was 1.7–8.5 cm. In general, most of the masses were clearly defined, the section was grey red and grey yellow, and the focus was grey white. Individual cases were accompanied by cystic changes. There were two cases of a tumour adjacent to the renal capsule that infiltrated the renal capsule (cases 5 and 6): one case of a tumour that infiltrated the fat of the renal hilum, with an intravenous tumour thrombus (case 3), and one case involved a leiomyomatous-like stroma (case 7). Under the microscope, RCC-like area and the haemangioblastoma-like area accounted for different proportions, with most being haemangioblastoma-like areas. In our case, the CCRCC-like area was the main area. There was a gradual transition zone between the two components, and only one case had no transition (case 3). Considering the existence of a transitional zone (6/7) between the two components of this tumour, they might coexpress vimentin, CAIX, PAX8, PAX2, and HIF1α; however, they had their own characteristics, suggesting that these components are two morphological manifestations of the same tumour and not a simple collision between renal cell carcinoma and haemangioblastoma. The two components of the tumour had different proportions in different cases, especially when the haemangioblastoma-like components were the main components, due to positivity of CAIX, PAX2, and PAX8 in sporadic renal haemangioblastoma, which might cause a critical diagnostic pitfall in differential diagnosis from CCRCC [10]. To avoid missed diagnosis and misdiagnosis, we must pay attention to systemic evaluation, including macroscopic, microscopic, and immunohistochemical findings. In some cases, molecular genetic studies might be necessary. Among the reported 7 cases, 3 patients were subjected to gene sequencing (cases 5, 6, and 7) and 2 fluorescence in situ hybridization (FISH) detection; no *TFE3* or *TFEB* gene translocation was found, and no von Hippel–Lindau (*VHL*) gene mutations were found. Our case is the first discovery of genetic abnormalities, and *TSC2* and *SETD2* variations were found.

**Table 1:** Cases of haemangioblastoma-like RCCs reported in the literature

Case	Subtype	WHO/ISUP grade	Age	Sex	Position	Size (cm)	Gene	Operation mode	Clinical symptoms	Clinical history	Follow-up
1 [1]	CCRCC	Grade 1	49	Male	Right	3.5	NA	Partial nephrectomy	NA	None	36 months
2 [1]	CCRCC	Grade 1	32	Female	Right	3.2	NA	Total nephrectomy	NA	None	36 months
3 [2]	CCRCC	NA	69	Male	Left	4	NA	Total nephrectomy	Haematuria and clots	Carcinoma of the bladder	NA
4 [3]	CCPRCC	Grade 2 with focal 3	75	Female	Left	1.7	NA	Partial nephrectomy	Routine physical examination	None	6 months
5 [4]	CCRCC	NA	33	Female	Right	4	No TFE3 or TFEB gene translocation	Total nephrectomy	Routine physical examination	None	34 months
6 [4]	CCRCC	NA	66	Female	Left	8.5	No TFE3 or TFEB gene translocation, no VHL gene isolation	Total nephrectomy	Routine physical examination	None	40 months
7^present^	CCRCC	Grades 1–2	38	Female	Right	2	TSC2 and SETD2 variations	Partial nephrectomy	Discomfort in the right kidney	Lung cancer	18 months

*CCRCC* clear cell renal cell carcinoma, *CCPRCC* clear cell papillary renal cell carcinoma, *NA* not available

Tuberous sclerosis complex (TSC) is a multisystem hereditary disorder affecting multiple organs, including the brain, heart, kidney, lung (lymphangioleiomyomatosis), and skin [11]. There are three major renal lesions in TSC: angiomyolipomas, cysts, and RCC. Two studies have summarized the clinical and pathologic features of TSC-associated renal cell carcinoma (TSC-RCC). Guo et al. [12] studied 57 RCCs from 18 TSC patients (13 females and 5 males). The 57 RCCs exhibited 3 major distinct morphologies, as follows:

1. Thirty percent of cases had features similar to tumours previously described as “renal angiomyoadenomatous tumour” or “RCC with smooth muscle stroma.”
2. Fifty-nine percent of cases showed features similar to chromophobe RCC.
3. Eleven percent of cases showed a granular eosinophilic-macrocystic morphology.

The mean age at the time of surgery was 42 years (range 7–65 years). No distant metastatic disease had occurred in the 15 patients for whom follow-up was available. Yang et al. [13] studied 19 TSC-RCC patients, and no distant metastasis had occurred in the 14 patients for whom follow-up was available. The conclusions from the two studies were female predominance and good prognosis in most TSC-RCC cases.

RCCLMS is an emerging subtype of RCC. Shah et al. studied 23 RCCLMS cases [14]. The patients had a mean age of 52 years, with a 2:1 female predominance and an average tumour size of 2.3 cm. All patients presented with a solitary renal mass that was incidentally detected during a clinical workup for haematuria or by imaging studies for other symptoms. Microscopically, all (18 [100%]) tumours had a low-power nodular architecture with the epithelium comprising elongated tubules with frequent branching, lined by cells with a voluminous clear to mildly eosinophilic cytoplasm, and separated by a variable smooth muscle rich stroma. The majority (16 [89%]) of the tumours showed thick fibromuscular tissue (pseudocapsule) at the periphery. A biphasic pattern of collapsed acini that surrounded the tubules with voluminous cytoplasm (7 [39%]), a focally prominent papillary architecture (6 [33%]), peritumoural lymphoid aggregates (6 [33%]), and haemosiderin-laden macrophages (6 [33%]) were also frequently found. The tumours were characterized by diffuse CK7 expression, diffuse CAIX expression in a membranous pattern (8/11 [73%]), diffuse cytoplasmic CD10 expression (8/8 [100%]), diffuse cytoplasmic CAM5.2 expression (11/11 [100%]), and variable cytoplasmic desmin reactivity in the smooth muscle component (10/10 [100%]). *TSC2* mutation was detected in 4 cases. The histological morphology of some of our cases was consistent with this study. The current case was mainly characterized by acinar structure without papillary structure, and immunohistochemical CD10 expression was negative.

*TSC1* and *TSC2* mutations also occur in sporadic RCC, including CCRCC, ChRCC, unclassified eosinophilic RCC, RCCLMS, eosinophilic solid and cystic renal cell carcinoma (ESC-RCC), hybrid oncocytic/chromophobe tumour (HOCT) and chromophobe-like RCC, low-grade oncocytic tumour (LOT) of the kidney, and eosinophilic vacuolated tumour (EVT), though at a low frequency [11]. The morphology of these tumours is similar to that of RCC associated with TSC.

Our case previously had lung microinvasive adenocarcinoma. There was no other treatment except regular follow-up after the operation, and to date, there has been no recurrence or metastasis, and no obvious abnormality was found in the right lung and other lobes of the left lung. In the kidney, no cysts or angioleiomyomas were found except for the tumour. Therefore, we believe that although *TSC2* gene variation was detected in this case, it did not meet the diagnostic criteria of tuberous sclerosis. Our case was sporadic renal cell carcinoma with *TSC2* variation.

Interestingly, although our case involved haemangioblastoma-like regions, immunohistochemistry also detected expression of the HIF pathway-related proteins HIF1α and CAIX, but no *VHL* gene change was detected. Instead, *SETD2* gene variation was detected. *SETD2* is located at 3p21 and the *VHL* gene at 3p25. Functional loss of *VHL* results in impaired ubiquitylation and accumulation of hypoxia-inducible factors (HIFs) within cell nuclei. Accumulated HIFs, in turn, increase production of several growth factors that have key roles in RCC progression [15]. *SETD2* and *VHL* genes are both located on chromosome 3 and in close proximity. According to the genetic and morphological changes in our case, we speculate that interaction between these genes occurs, but further confirmation is needed. Mutation of the histone H3 lysine 36 histone (H3K36) methyltransferase gene *SETD2* may produce dysfunction in corresponding tumour tissue proteins, leading to tumorigenesis, progression, chemotherapy resistance, and unfavourable prognosis [16]. However, in the setting of metastatic disease, next-generation sequencing (NGS) data failed to demonstrate a relationship between overall survival and *SETD2* mutation status. The role of *SETD2* mutation in metastatic disease therefore remains to be fully elucidated [15].

In conclusion, this paper aimed to emphasize the clinicopathological characteristics of rare tumours and help pathologists and clinicians to better understand this disease. In addition to one case of RCC with haemangioblastoma-like features reported in the literature, no recurrence or metastasis after surgery has been reported for other RCCs with haemangioblastoma-like features and RCCs with leiomyomatous stroma. Our case showed the characteristics of haemangioblastoma-like and leiomyomatous stroma harbouring *TSC2* and *SETD2* variations without treatment after the operation. After 18 months of follow-up, no recurrence or metastasis occurred. Because of the special morphological characteristics of this tumour, whether it is a new tumour entity or a variant of CCRCC remains to be determined. Its biological behaviour and prognosis need to be observed for a long time.

## Data Availability

Records and data pertaining to the case are in the patient’s secure medical records in the Second Affiliated Hospital of Dalian Medical University. All searched data by literature review are included in this paper.

## Abbreviations

WHO: World Health Organization
RCC: Renal cell carcinoma
CCRCC: Clear cell renal cell carcinoma
CCPRCC: Clear cell papillary renal cell carcinoma
RCCLMS: Renal cell carcinoma with (angio)leiomyomatous stroma
ChRCC: Chromophobe renal cell carcinoma
ESC-RCC: Eosinophilic solid and cystic renal cell carcinoma
HOCT: Hybrid oncocytic/chromophobe tumour
LOT: Low-grade oncocytic tumour
EVT: Eosinophilic vacuolated tumour
TSC: Tuberous sclerosis complex
TSC-RCC: TSC-associated renal cell carcinoma
WES: Whole-exon sequencing
SNVs: Single-nucleotide variations
VHL: Von Hippel–Lindau
NGS: Next-generation sequencing
